# A New Approach to Recover Bioactive Compounds from Apple Pomace: Healthy Jelly Candies

**DOI:** 10.3390/foods14010039

**Published:** 2024-12-27

**Authors:** Liliana Ciurlă, Iuliana-Maria Enache, Ioana Buțerchi, Gabriela Mihalache, Florin Daniel Lipșa, Antoanela Patraș

**Affiliations:** 1 Faculty of Horticulture, “Ion Ionescu de la Brad” Iasi University of Life Sciences, 3 Mihail Sadoveanu, Alley, 700490 Iasi, Romania; liliana.ciurla@iuls.ro (L.C.);; 2 Faculty of Agriculture, “Ion Ionescu de la Brad” Iasi University of Life Sciences, 3 Mihail Sadoveanu, Alley, 700490 Iasi, Romania; 3 Integrated Centre of Environmental Science Studies in the North Eastern Region (CERNESIM), “Alexandru Ioan Cuza” University of Iasi, 11 Carol I, 700506 Iasi, Romania

**Keywords:** apple pomace, bioactive compounds, jelly candy, functional food, sustainability

## Abstract

Rich in bioactive compounds, carbohydrates, fibers, minerals, and trace elements, apple pomace (AP) is a significant agro-industrial by-product, which pollutes and brings high management costs. The current study investigates the possibility of using an aqueous AP extract (APE) as the main ingredient in a jelly candy recipe, replacing artificial colors and flavors and improving its nutritional value. APE and formulated jelly candies were analyzed in terms of their phytochemical profile, antioxidant capacity, and color parameters. In addition, the microbiological and sensory properties of the jelly candies, as well as their behavior during storage, were analyzed. An HPLC analysis of AP revealed the presence of 9 individual phenolic compounds, with a high content of protocatechuic (375.21 ± 18.76 µg/g DW) and *p*-hydroxybenzoic (164.96 ± 13.83 µg/g DW) acids. The results of this study prove the presence of bioactive compounds with antioxidant and antidiabetic properties in both APE and its candies. Investigation on jelly candies with APE revealed an antioxidant capacity of 142.03 ± 1.08 mmol TE/g DW and a total polyphenolic content of 8.25 ± 0.17 mg GAE/g DW. Additionally, a sensory analysis highly appreciated the proposed jelly with APE, with scores higher than 4.70/5.00 for all evaluated attributes. Thus, this study succeeded in developing a new approach to recovering bioactive compounds from AP, demonstrating the potential of this by-product to improve jelly candies’ attributes while promoting sustainability through waste reduction and the effective use of natural resources.

## 1. Introduction

In the context of rapid demographic growth and, with it, consumption, there is also an increase in waste resulting from the agro-food industry [1]. This large amount of industrial agro-food by-products has attracted attention because of the negative environmental implications and the high costs involved in their disposal [2]. Consequently, sustainable solutions are needed for their exploitation [2,3]. The United Nations Sustainable Development Goals have encouraged current research to focus on the recovery of agri-food by-products and their potential as functional and nutritional ingredients. This strategy aims to ensure sustainable patterns of consumption and production through *Goal 12—Responsible consumption and production,* consistent with achieving good health and well-being [4].

Apple pomace (AP), a by-product of apple processing, stands out among the many agro-food industrial wastes found globally. This is due to the widespread consumption of apples in diets across different cultures, likely owing to their appealing taste, convenience, and year-round availability [3]. Regarding Romania, apples are the most consumed fruits, with a consumption of 5.4 kg per capita in 2018, according to the Romanian National Institute of Statistics [5]. Every year, a significant quantity of apples is utilized worldwide in the production of juices, ciders, and concentrates, resulting in the generation of substantial residues estimated at around 4 million tons [6]. Nevertheless, AP is an extraordinary resource of bioactive compounds with health-beneficial properties, such as dietary fiber, carbohydrates, carotenoids, polyphenols, vitamins, minerals, and pectin [7,8,9,10]. A literature review highlighted the antioxidant, anti-inflammatory, antibacterial, and antiviral activity of this by-product [11]. The antioxidant capacity of AP is mainly due to its phenolic content [12]. More than 82% of the phenolic compounds found in apples remain in the pomace after processing [13,14]. In addition to the polyphenols, other bioactive compounds, e.g., vitamins C and E (seeds), as well as carotenoids, contribute to AP’s antioxidant capacity [12].

The extraction of the above-mentioned bioactive compounds from waste or by-products represents one of the specific issues of biotechnology. These compounds play an important role through apple pomace valorization [15,16,17]. Previous studies concerning the recovery and valorization of AP led to the production of organic acids, aroma compounds, bioethanol, enzymes, edible mushrooms, edible fibers, pectin recovery, natural antioxidants, protein-enriched animal feed, etc. [18]. Also, the use of AP as a natural additive aligns with current trends in food science and technology. Fruits, vegetables, or their by-products, such as AP, represent an inexpensive source of colorants, antioxidants, antimicrobials, and sweeteners [19]. For example, apple carotenoids (found mainly in peels), in addition to their antioxidant capacity, are also natural pigments (lutein, violaxanthin, neoxanthin, and β-carotene) [20], and the polyphenols from AP represent natural antioxidant and antimicrobial agents [19]. As a result of the increasing interest in this by-product, important research studies were carried out on the inclusion of AP in new fortified food products. Bakery products with AP, e.g., biscuits [21], bread [2,22], or cookies [23,24], were prepared, aiming to improve fiber content and nutrient intake and to reduce saturated fat content. AP was also successfully used as an antimicrobial, flavoring, coloring, and thickening agent in meat products, such as chicken sausages [25,26] and patties [8], buffalo meat sausages [27], or mutton meat [28]. It has been observed that the inclusion of AP in meat products leads to a change in the color of the product, which was positively assessed in a sensory analysis [11]. Yogurt with AP [3,4] showed improved fiber content and antioxidant properties. Due to the large amounts of flavor-enhancing compounds and soluble fibers, such as pectin compounds, AP has been used in confectionery preparation [29], e.g., jellies with AP and quince [30] and jellies with AP flour [31].

Considering the above-mentioned, the main aim of this study was to recover bioactive compounds from AP and to incorporate them into a new recipe for healthy jelly candies. Over time, jelly candy consumption around the world has increased, especially commercial jellies, which are rich in sugar, synthetic colorants, and preservatives. Unfortunately, all of them are associated with important human body damage (diabetes, cardiovascular and gastrointestinal disorders, etc.) [32]. This study proposes a new, simple, clean recipe for the preparation of jelly candies that are sugar-free, with a natural color and flavor, and that originated from AP. Moreover, the high content in bioactive compounds of the raw material (AP) leads to an enriched, nutraceutical product, with health-protecting properties. The original elements of the jelly candy product are represented by the natural origin of the ingredients (there are no synthetic colorants and no synthetic preservatives), the small number of ingredients, a low production cost, and the use of an agro-industrial by-product (AP) as raw material.

## 2. Materials and Methods

### 2.1. Chemicals and Reagents

The extraction of bioactive compounds from AP was performed using potable water as a solvent. The total antioxidant capacity was analyzed using methanol, 2,2-Diphenyl-1-picrylhydrazyl (DPPH), and 6-Hydroxy-2.5.7.8-tetramethylchroman-2-carboxylic acid (Trolox). In order to determine the total polyphenol content, the Folin–Ciocalteu reagent, sodium carbonate, and gallic acid were used; for the titratable acidity, NaOH was used; for the biological activity of the samples, *p*-nitrophenyl-α-D-glucopyranoside, α-amylase, and β-glucosidase were employed. The following HPLC-grade reagents were utilized for an HPLC analysis: gallic acid, protocatechuic acid, *p*-hydroxybenzoic acid, vanillic acid, caffeic acid, catechin, chlorogenic acid, vanillin, syringic acid, coumaric acid, epicatechin, ferulic acid, salicylic acid, sinapic acid, resveratrol, and quercetin. The reagents were purchased from Sigma-Aldrich (Steinheim, Germany). For microbiological studies, the following were used: sterile peptone water; Rapid’E.coli 2 agar (Bio-Rad Laboratories, Marnes-la-Coquette, France); and Potato Dextrose Agar (Scharlau, Barcelona, Spain). All the reagents used in this study were of analytical grade.

### 2.2. Bioactive Compounds’ Extraction from Apple Pomace

The apple pomace analyzed in the present research was provided by SC SILVER ROM AGRO SRL Iasi, a local natural juice producer. In this production batch, a random mixture of the following apple varieties at full maturity were used: Idared, Florina, Golden Delicious, and Generos. The fresh apple pomace was dried at 40 °C, using a convective laboratory oven (BIOBASE BOV-T30C, Jinan, China), up to constant weight (with the KERN-ADB 100-4 weighing scale, 120 g, Balingen, Germany), in order to be stabilized. The dry material was ground using a laboratory grinder mill (MRC CUP-300CC, Harlow, UK) up to 100 µm and stored in a dark, dry, and cool place prior to jelly preparation. The extraction of the bioactive compounds was carried out with potable water (ratio 1:5) in an ultrasonic water bath (HBM GL Serie 2.5 Liter Ultrasoon reiniger, Moordrecht, The Netherlands) at 40 °C for 30 min. The obtained mixture was filtered, and the clear liquid, namely the apple pomace extract (APE), was used to prepare jellies.

### 2.3. Apple Pomace Characterization

The apple pomace was analyzed regarding its physicochemical characteristics, color parameters, the content of total polyphenolic compounds, carotenoid profile, individual phenolic compounds, antioxidant capacity, and antidiabetic potential.

#### 2.3.1. Physicochemical Analysis of the Apple Pomace Extract

The soluble solids content (TSS) of the APE, as prepared in Section 2.2, was evaluated using a refractometer (Optika HR-150N, Ponteranica, Italy), and the results are expressed in °Brix (°Bx). The total dry matter (%) was determined following the gravimetric method. The titrimetric method was used for titratable acidity (TA) determination. Briefly, the APE was homogenized with distilled water and titrated with a NaOH standard solution. The results are expressed in % malic acid. The pH was measured with a laboratory pH meter (Testo 206-pH2, Lenzkirch, Germany), with the results being expressed in pH units [33].

#### 2.3.2. Antioxidant Capacity of the Apple Pomace Extract

An analysis of the antioxidant capacity of APE was performed directly, without further processing. Briefly, a 100 µL aliquot of APE was mixed with 3900 µL of the DPPH reagent (0.1 M) [34]. Meanwhile, a blank sample was prepared by replacing the aliquot with methanol. After 90 min, the absorbance was read by T70 a UV-Vis spectrophotometer (PG Instruments Ltd., Alma Park, United Kingdom) at 515 nm. The obtained results are expressed as mmol Trolox equivalents/g dry weight of APE (mmol TE/g DW).

#### 2.3.3. Total Polyphenolic Content of the Apple Pomace Extract

An analysis of the total polyphenol content (TPC) of APE was carried out by using the Folin–Ciocalteu (FC) method with some modifications [35]. Briefly, 100 µL of the aliquot of APE was mixed with 7900 µL of distillated water, 500 µL of the FC reagent, and 1500 µL of sodium carbonate (20%). After 60 min of incubation in dark conditions, the absorbance of the sample was read at 760 nm. The obtained results for TPC are expressed as mg gallic acid equivalents/g dry weight of APE (mg GAE/g DW).

#### 2.3.4. Evaluation of the Carotenoid Profile of Apple Pomace

An evaluation of the carotenoid profile (total carotenoids, lycopene, and β-carotene content) was performed by a UV-Vis spectrophotometric analysis, using the Britton equation, according to the protocol previously described by Britton et al. (1995) [36]. A sample of 2.5 g of apple pomace was extracted with 50 mL of hexane, as follows: the sample was ground in a mortar in the presence of 10 mL of hexane. The liquid fraction was collected, and the operation was repeated 4 more times for the same solid fraction with another 10 mL volume of hexane. The hexane fractions were mixed and centrifuged at 5000× *g* and 4 °C for 10 min, and the absorbance of the supernatant was read at λ = 470 nm for the total carotenoids, λ = 450 nm for β-carotene, and λ = 503 nm for lycopene.

#### 2.3.5. Chromatographic Analysis of the Apple Pomace

Previously reported chromatographic conditions [37] were used for the identification and quantification of individual phenolic compounds in apple pomace. For the extraction of phenolic compounds, AP was mixed with an extraction solvent (50% methanol, containing 0.3% hydrochloric acid) in a ratio of 1:4 (solid:solvent), then magnetically stirred for 30 min in a water bath at 45 °C. The resulting mixture was centrifuged at 5000× *g* and 4 °C for 30 min. A total of 8 mL of the collected clear supernatant were concentrated on a water bath at 45 °C for 1.5 h and then diluted to 5 mL with the same solvent. The obtained solution was filtered through a 0.45 µm membrane before injection into the HPLC system.

#### 2.3.6. Colorimetric Analysis of the Apple Pomace Extract

The color parameters of the APE were assessed using the colorimeter MiniScan XE Plus and accompanying software (Reston, VA, USA), which was standardized before each analysis, according to the device’s specifications. In summary, the device screen displays three parameters (L*—lightness, a*—red/green color components, and b*—yellow/blue color components). Based on the obtained values, three additional factors were calculated [38,39]:

- Chroma (c* = (a*^2^ + b*^2^)^1/2^) indicates chromaticness, which is a measurement of color intensity or saturation that varies from 0 (totally unsaturated) to 100 or above (pure color);
- The hue angle (h* = arctan(b*/a*) represents the tone and is expressed in degrees on a 360° scale;
- The overall colorimetric difference (∆E* = (∆L^2^ + ∆a^2^ + ∆b^2^)^1/2^) shows the global change in color of a sample compared to another one.

#### 2.3.7. Biological Activity of Apple Pomace Extract

The antidiabetic potential of APE was demonstrated by the inhibitory activity of two metabolically important enzymes, α-amylase and β-glucosidase, using a methodology previously described [40,41]. The inhibition rate of α-amylase by the APE was measured according to Costamagna et al. [40], with slight modifications. Briefly, a volume of 100 µL of extract solutions (0.5, 1, and 5 µg/mL concentrations of the extract diluted in ultrapure water) was added to 100 µL of an α-amylase solution (1 mg/mL in 0.1 M of a phosphate buffer solution, pH = 6.9). After 5 min of incubation at room temperature, 100 µL of a 1% (*w*/*v*) starch solution in distilled water was added to the reaction mixture and incubated for another 20 min at 37 °C. Further, 200 µL of 0.04 M of a 3,5-dinitrosalicylic acid (DNS) reagent was added to the reaction mixture, followed by heating at 100 °C for 5 min in a thermostatic water bath. Finally, the samples were diluted with 2 mL of distilled water, and the absorbance was measured at 540 nm with a UV-Vis spectrophotometer. The β-glucosidase inhibitory activity of the APE was also measured according to Costamagna et al. [40]. The reaction mixture contained 50 µL of a β-glucosidase solution (1 mg/mL in 0.1 M of a phosphate buffer solution, pH = 6.9) and 50 µL of extract solutions (0.5, 1, and 5 µg/mL). After pre-incubation of the reaction mixture at room temperature for 5 min, the enzyme reaction started by adding 50 µL of 25 mM of *p*-nitrophenyl-α-D-glucopyranoside and 1.6 mL of 0.1 M of a phosphate buffer solution (pH = 6.9). The mixture was incubated for 15 min at 37 °C. Then, 800 µL of 0.2 M of sodium carbonate was added. The absorbance was read at 405 nm with a UV-Vis spectrophotometer. The results represent the inhibition ratio of the enzyme (%) and are expressed as the mean of three replicates.

### 2.4. Jelly Candies’ Formulation

**Figure 1:**
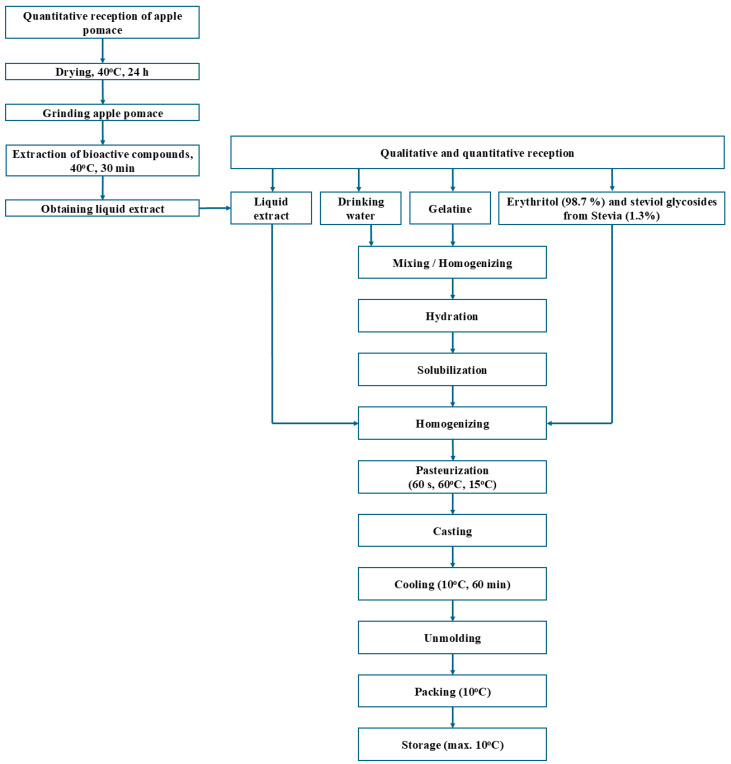
Technological process of jelly candy formulation.

The preparation of the enriched jelly candies was realized following the steps presented in the scheme of the technological process (Figure 1). To obtain 1 kg of jelly candies, the following were used: 452.49 mL of APE, 452.49 mL of potable water, 67.87 g of gelatin, and 45.24 g of a mixture of erythritol (98.7%) and steviol glycosides from stevia (1.3%). The preparation process began with a qualitative and quantitative reception of all ingredients, followed by their dosage. In the next step, the gelatin was hydrated with potable water for 10 min at room temperature. Then, all the ingredients were mixed, homogenized, and pasteurized (60 sec at 60 °C) in a water bath (Raypa BAD-4, 12 L, Barcelona, Spain). This process continued with a casting (distribution) of the resulting mixture into molds, followed by cooling (60 min at 10 °C) in a refrigerator (POL-EKO, CHL 3, Wodzislaw Slaski, Poland) and unmolding. In the last step, the jelly candies were packed, then stored at 10 °C.

The control sample of jellies was prepared following the above recipe but replacing the APE with water. More precisely, for the preparation of 1 kg of jelly candies without APE, the following were used: 904.98 mL of potable water, 67.87 g of gelatin, and 45.24 g of a mixture of erythritol (98.7%) and steviol glycosides from stevia (1.3%). A laboratory size batch of 1 kg was prepared for each type of jelly candy.

### 2.5. Jelly Candies’ Analysis

#### 2.5.1. Physicochemical Analysis, Total Polyphenolic Content, and Antioxidant Capacity

Pre-analysis processing was performed by mixing the jelly candies with distilled water (solid-liquid ratio = 1:5) until complete homogenization, and the clear liquid separated by centrifugation was used to analyze the physicochemical parameters, TPC, and antioxidant capacity, following the techniques described in Section 2.3.1, Section 2.3.2 and Section 2.3.3

#### 2.5.2. Carotenoid Profile of Jelly Candies with Apple Pomace Extract

The carotenoid profile of jelly candies with APE was assessed as described in Section 2.3.4, similar to the analysis of apple pomace, and a sample of 2.5 g of jellies with APE was used instead of 2.5 g of AP.

#### 2.5.3. A Colorimetric Analysis of the Jelly Candies

The color parameters of the jelly candies with and without APE were directly assessed without further preparation, as described for APE in Section 2.3.6.

### 2.6. Biological Activity of Jelly Candies

The antidiabetic potential of jelly candies with APE was evaluated using the methodology indicated for APE in Section 2.3.7 [40,41].

### 2.7. Microbiological Studies

The presence of enterobacteria, yeasts, and fungi was determined in 1 g of the product immediately after processing (0 days = T0) and at 2 (T2), 5 (T5), and 7 days (T7) of storage at 4 °C. The samples were homogenized into 9 mL of sterile peptone water, and decimal dilutions were performed (Teixeira-Lemos et al., 2021). The determination of *Escherichia coli* and other coliforms was performed according to ISO 16140 on Rapid’E.coli 2 agar (Bio-Rad Laboratories, Marnes-la-Coquette, France) after incubating the plates for 24 h at 37 °C (ISO 16140-3:2021) [42]. Yeasts and fungi were counted by plating the samples on Potato Dextrose Agar (Scharlau, Barcelona, Spain) and incubating them for 5 days at 28 °C [43]. Samples without apple pomace were used as controls. The results are expressed as log colony-forming units per gram of product (log CFU/g).

### 2.8. Sensory Analysis

A sensory analysis of jelly candies with APE and the control sample—jelly candies without APE—was carried out with 40 panelists previously trained on how to assess the organoleptic qualities of the tested products. It should be noted that all the panelists have a higher education degree and come from urban areas, and 80% of them are non-smokers. Thus, the panelists (20 women and 20 men), aged between 30–55 years old, had the task of evaluating the samples mentioned above in terms of appearance and shape, consistency, color, aroma, and taste. Using a 5-point hedonic scale, panel members evaluated the product’s appearance, color, smell, and taste: 1—dislike greatly; 2—dislike somewhat; 3—neither like nor dislike; 4—like slightly; 5—like highly.

### 2.9. Storage Stability

At the same time with the microbiological studies (T0, T2, T5, and T7), the color parameters, the phytochemical compounds (polyphenol and carotenoid content), and the antioxidant capacity of jelly candies with and without APE were tested.

### 2.10. Statistical Analysis of Data

In this study, all the analyses were performed in triplicate, and the results are expressed as a mean ± standard deviation. The standard deviations were calculated with the Microsoft Excel 2010 software. The results were compared using the IBM SPSS Statistics 20 software, one-way ANOVA, and Tukey’s post-hoc multiple comparison test. The significance level was 0.05.

## 3. Results and Discussion

### 3.1. Phytochemical Characterisation of Apple Pomace

Prior to manufacturing the jelly candies, the AP/APE introduced into the jellies was analyzed for its physicochemical and color properties, as well as its antidiabetic potential. The obtained results are presented in Table 1.

APE registered a pH value of 3.20 ± 0.10, consistent with the value of total acidity, 0.46 ± 0.02%. These findings may be associated with naturally occurring organic acids in AP, e.g., malic acid [3]. Numerous studies [3,44,45] that focused on AP reported good results regarding the antioxidant capacity of this plant material. Consistent with these studies, we obtained a significant value of 193.26 ± 0.24 mmol TE/g DW for the aqueous extract. This result aligns with the important concentrations of bioactive substances present in apple pomace. It is well documented that AP is an important source of phenolic compounds, with its antioxidant potential being considered a consequence of its polyphenol content [10,46,47]. In the current study, a remarkable value of 26.94 ± 3.76 mg GAE/g DW was found for APE. In addition to polyphenol content, carotenoids also contribute to the antioxidant capacity of AP. The investigation on the carotenoid profile of APE revealed a total carotenoid content of 10.32 ± 0.12 µg/g DW, a β-carotene content of 4.58 ± 0.08 µg /g DW, and a lycopene concentration of 4.54 ± 0.04 µg /g DW. A higher content of total carotenoids (4.93 mg/100 g DW) was found by Popescu et al. (2022) for apple pomace obtained from a Golden Delicious cultivar [48]. Apple pomace from Gala, Fuji, and Granny varieties, studied by Salari et al. (2024), recorded β-carotene values ranging from 0.3 to 0.7 mg/100 g DW, following an HPLC determination [49]. An LC–MS study regarding the carotenoid compositions of the peel of 13 commercial apple cultivars revealed a content of all-trans-β-carotene ranging from 1.62 ± 0.04 to 2.51 ± 0.09 µg/g DW for yellow-skinned apples, from 1.49 ± 0.27 to 5.44 ± 0.40 µg/g DW for red-skinned apples, and from 3.23 ± 0.18 to 30.31 ± 1.56 µg/g DW for green-skinned apples. The same study highlighted that the total pigment content in the peels was higher than in the flesh for all studied cultivars [20]. The measured color parameters for APE indicated a lightness of 40.38 ± 0.65, shades of red due to the positive value of the parameter a* (21.21 ± 0.34), and shades of yellow due to the positive value of the parameter b* (20.51 ± 0.28). The investigation on the antidiabetic potential of APE displayed similar inhibitory activity against the two studied enzymes, with an inhibition ratio of 32.96 ± 1.72% for α-amylase and 33.32 ± 1.59% for β-glucosidase.

All the above presented results emphasize the potential of APE to be used as a natural food additive.

### 3.2. Chromatographic Analysis of Apple Pomace

Phenolic compounds significantly influence antioxidant capacity and are also responsible for the beneficial effects of apple consumption [50]. Following the HPLC analysis of AP, among the 16 phenolic compounds investigated, nine were identified, namely gallic acid, protocatechuic acid, *p*-hydroxybenzoic acid, vanillic acid, catechin, chlorogenic acid, vanillin, coumaric acid, and salicylic acid. The content of each identified phenolic compound, expressed as µg/g DW ± standard deviation, is presented in Table 2.

As can be observed, the highest concentrations were found for protocatechuic acid and *p*-hydroxybenzoic acid. These high amounts may be the result of the acidic conditions used for extraction, as indicated in a previous study [51], which showed that apple pomace subjected to acid hydrolysis released mainly *p*-hydroxybenzoic acid and protocatechuic acid. Protocatechuic acid is a polyphenol widely distributed in plants, edible fruits, and vegetables, playing a role in self-defense. The inclusion of this compound in one’s diet is associated with a reduced risk of developing chronic diseases (such as cardiovascular and neurodegenerative diseases, diabetes, obesity, etc.); a beneficial effect on chronic inflammation through its antioxidant capacity; and a positive impact on intestinal microbiota. Furthermore, the chemopreventive potential of protocatechuic acid was demonstrated in vitro [52]. Various biological activities, i.e., antimicrobial, hypoglycemic, anti-inflammatory, antiplatelet, nematicide, antiviral, antioxidant, etc., were also reported for *p*-hydroxybenzoic acid [53].

A significant amount of salicylic acid (94.91 ± 0.65 µg/g DW) was observed during this analysis. Similarly, in a study by Hammad et al. (2021) [54], salicylic acid was found among the phenolic acids present in the highest concentration in an apple pomace extract. Salicylic acid is another polyphenol with a defensive role in plants and with health benefits for the human body, like antidiabetic, anticancer, antiviral, and anti-inflammatory effects [55].

For chlorogenic acid, a concentration of 54.03 ± 4.38 µg/g DW was registered. This phenolic acid is one of the major phenolic compounds found in apples [56], and its presence in apple pomace was demonstrated by other HPLC-DAD analyses [51,54,56,57,58,59,60]. Moreover, Rana et al. (2021) [59] found chlorogenic acid in five different cultivars of apple pomace and Hobbi et al. (2023) [57] in all the fractions of the studied apple pomace. The health benefits of the consumption of chlorogenic acid are correlated with a lower risk of metabolic syndromes and chronic diseases [61].

Catechin was found in lower amounts of 37.72 ± 2.84 µg/g DW, probably due to its limited presence in seeds and cores, as observed in a report of Hobbi et al. (2023) [57]. The lowest concentrations of the current study were obtained for gallic acid, vanillin, coumaric acid, and vanillic acid. A minor amount of gallic acid in apple pomace was also found by Oldoni et al. (2020) [62], of coumaric acid by Rana et al. (2014) [58], and of vanillin by Hammad et al. (2021) [54]. Du et al. (2019) [60] identified vanillic acid in apple peels and pulps.

### 3.3. Manufacturing of Jelly Candies

The proposed recipe for jellies, based on liquid APE, aims to valorize the bioactive compounds of AP by reintroducing them into a simple food matrix. Thus, a clean recipe for jelly candies was developed, with a reduced number of ingredients, of natural origin. The main ingredient of this recipe is the aqueous extract of apple pomace resulting from the processing of apples into juice. The recovery of biologically active compounds from AP was carried out by aqueous extraction, avoiding toxic or potentially toxic solvents for the human body and the environment. The inclusion of this ingredient in the recipe was intended to replace synthetic artificial food dyes and flavors. Most jelly candies are prepared with synthetic dyes, such as azorubine (E 122), which have possible effects on human health, including allergic reactions, an intensification of asthma symptoms, intolerance in people sensitive to salicylates, and hyperactivity in children, and are suspected of carcinogenic properties (connected to urinary bladder cancer) [63,64]. The amount of extract introduced into the recipe was estimated using a preliminary sensory analysis. A variety of jellies containing different quantities of APE were tested, and the most appreciated one was considered for our study. Also, the sugar content of classic jellies limits their consumption for certain categories of people, such as diabetics or overweight people. For these reasons, in the proposed formulation, the sugar was replaced by a mixture of erythritol (98.7%) and steviol glycosides from stevia (1.3%), a zero-calorie sweetener approved as safe by The European Food Safety Authority (EFSA), which exerts no effects on blood glucose levels [65]. Thus, the prepared jelly candies with APE can be consumed by all categories of consumers, including those with sugar restrictions, diabetics, or overweight people. For the gelation of the food product, gelatin was chosen, a safe, low-cost, low-calorie natural ingredient and a source of essential amino acids [66,67]. Pasteurization was carried out at temperatures of 60 °C, for a short time, to avoid structural changes of gelatin and degradation of bioactive compounds.

**Figure 2:**
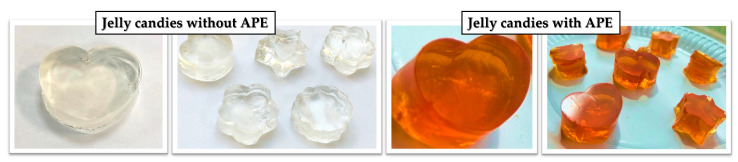
Prepared jelly candies with and without APE.

In order to ensure a proper comparison that highlights the improvements that APE brings to jellies (bioactive compounds, antioxidants, antidiabetic and antimicrobial potentials, as well as sensory attributes), a control jelly sample was prepared by replacing APE with water, without any other change, avoiding any interaction between ingredients and following the main idea of using as few ingredients as possible. For the preparation of the control jellies, the same amount of gelatin and sugar substitutes (a blend of 98.7% erythritol and 1.3% steviol glycosides from stevia) was used. No synthetic dye was utilized for the control jellies, as it was already established that jellies incorporating such additives (e.g., azorubine) exhibit prooxidant activity and other negative effects, as evidenced in a previous study comparing the properties of jelly candies with chokeberry extract and those with azorubine [64]. The jellies prepared with and without APE (Figure 2) were evaluated from the point of view of their physicochemical and microbiological characteristics, their antidiabetic potential, and their sensory attributes.

### 3.4. A Physicochemical Characterization of the Jelly Candies

The prepared jelly candies were tested for their bioactive compounds (polyphenol content, total carotenoids, β-carotene, and lycopene); antioxidant capacity; pH; soluble and total dry matter; and total acidity. The outcomes are shown in Table 3.

Regarding the pH of the analyzed samples, the results were 3.75 ± 0.25 for the jellies with APE and 4.04 ± 0.01 for jellies without APE, with the difference being statistically insignificant. The obtained values fall within the recommended pH range for gelatin-based products, 3.0–4.5, with lower values being associated with instability or the stoppage of gel formation [68]. Titratable acidity quantifies the overall acid concentration in a food product, which is usually given by organic acids. As organic acids found in food significantly impact flavor, color, or microbial stability, titratable acidity is considered a quality indicator of a product [69]. Consistent with the results obtained for pH, a higher value for titratable acidity was recorded for jelly candies with APE (0.33 ± 0.01%) and a lower value for jelly candies without APE (0.08 ± 0.02%). The value of titratable acidity obtained for jelly candies with APE is within the range of the titratable acidity obtained for the low-calorie jellies formulated by Zormand et al. (2022) [70]. Regarding soluble dry matter, the results obtained for the jellies with APE (19.00 ± 0.05°Bx) are lower than those generally reported for similar products due to the replacement of the sugar found in conventional jelly candies with a low glycemic index sweetener, namely the erythritol–stevia mixture [71]. Also, the reduced number of ingredients in the recipe led to a lower value of soluble dry matter.

The antioxidant potential of the apple pomace extract was successfully assimilated into the jelly product with APE, which registered a value of 142.03 ± 1.08 mmol TE/g DW. This result is significantly higher than the value obtained for the jelly product without APE (13.60 ± 0.58 mmol TE/g DW), highlighting the fact that apple pomace is an excellent source for improving the antioxidant capacity of a food product. The jelly candies prepared with apple pomace flour and gelatin by Gorjanovic et al. (2024) [31] also showed an important increase in the antioxidant capacity compared to a control sample without AP. Other previous fortification studies focus on the effects of adding AP to foods and also showed a considerable increase in antioxidant capacity [3,72].

The antioxidant capacity is influenced by the total polyphenolic content, which, for the proposed jelly candies with APE, revealed a significant value of 8.25 ± 0.17 mg GAE/g DW. The same effect of improving the total phenolic content of jellies has been shown by other authors when apple pomace flour was included in a gelatin jelly recipe [31]. The applied method measures not only the phenolic compounds with reducing properties but also other reducing compounds and can be used as an indicator for the total reducing capacity [73]. In fact, Folin–Ciocalteu, as well as DPPH and a few other methods (as the ferric-reducing antioxidant power, FRAP), are examples of electron transfer assays based on redox reactions, in which the antioxidants present in the sample transfer electrons to oxidants, such as DPPH or the metal ion of the Folin–Ciocalteu reagent [74].

Regarding the carotenoid profile of jelly candies with APE, a total carotenoid content of 3.61 ± 0.04 µg/g DW was obtained. The values obtained for lycopene and β-carotene were close to each other: 1.56 ± 0.04 µg of β-carotene/g DW and 1.84 ± 0.03 µg of lycopene/g DW, respectively. The carotenoid content of the jelly candies with APE was associated with the yellow color of the samples, as demonstrated by the results of the colorimetric study in Section 3.5.

### 3.5. Colorimetric Analysis of Jelly Candies

Table 4 displays the results of a comprehensive examination of the colorimetric profile of the enriched jelly candies with APE and the control sample—jelly candies without APE.

Color serves as a significant indicator of the quality and acceptance of food products [68]. Previous research has shown that the vibrant color of a dish is appetizing and associated with a perception of the freshness of food [75]. The L* parameter indicates the lightness of a sample and can vary from 0 (black) to 100 (white) [39]. The lightness of the jellies with APE (46.42 ± 0.11) was significantly lower than the lightness of the jellies without APE (74.57 ± 0.30). This significant difference marks a reduction in lightness as a result of the introduction of APE in the recipe.

The a* parameter represents the red/green color component, showing shades of red if positive and shades of green if negative [39,76]. The outcomes of our investigation show positive values for the a* parameters that indicate red color nuances for jelly candies with APE (22.79 ± 0.49). The a* parameter measured for the jelly candies without APE was also positive but significantly lower. The red color of food positively influences consumers, leading to an increased appetite [75]. The parameter a* can be correlated with polyphenolic content. Furthermore, the red shadow of the jelly candy with APE is also associated with the drying process of the raw material [77].

The b* parameter represents the yellow/blue color component, showing shades of yellow if positive and shades of blue if negative [39,76]. Furthermore, the b* parameter obtained for the jelly candies with APE had positive values (20.54 ± 0.23) which are associated with yellow hues [39,76]. The b* parameter values obtained for jelly candies without APE were also positive but significantly lower. This result correlates with that of the carotenoid content, shown in Section 3.4. Considering the values of the a* and b* parameters for jelly candies with APE, the color of the samples is inclined towards red and yellow shades.

The values of the parameters L*, a*, and b* obtained for APE and those obtained for jelly candies with APE are different but close to each other; thus, it can be stated that the jellies borrow the color of the extract. This result is in accordance with the Romanian regulation regarding jelly candies (Order 523/808/351/2003), which stipulates that the color of jellies must be characteristic of the juice used in their preparation [33].

The ΔE* parameter reflects the overall colorimetric difference between two samples. If ΔE* > 1, an observer can typically distinguish between two colors; however, due to various interferences, color discrimination can often be worse [39,78]. Color differences are regarded as small differences if ΔE* < 1.5, distinct if 1.5 < ΔE* < 3, and very distinct if ΔE* > 3 [39,79]. The overall colorimetric difference between jellies with and without APE has a high value of 31.29 ± 0.35, indicating a very distinct color difference, proving that APE is the source of the color of jellies with APE.

### 3.6. Biological Activity of Jelly Candies with APE

Diabetes and obesity are diseases with increasing prevalence in Romania and worldwide, with worrying statistics being published by FAOSTAT [80]. In view of this medical problem of our days, jelly candies with apple pomace extract have been formulated as a dessert alternative that does not endanger health. Inhibition of the enzymes involved in the digestion of carbohydrates, such as β-glucosidase and α-amylase [81], to prevent the absorption of glucose from food is a strategy used for the development of antidiabetic drugs [82]. These enzymes catalyze the cleavage of glycosidic bonds in polysaccharide chains to form smaller carbohydrate molecules, resulting in increased blood glucose levels [82,83]. Inhibitors targeting these enzymes can slow the release of glucose from the polysaccharide chains and delay its absorption into the bloodstream [84]. Natural inhibitors of these enzymes may represent an alternative with fewer side effects to synthetic drugs [82]. Considering this, the inhibitory effect of the proposed food product on β-glucosidase and α-amylase was analyzed to evaluate its antidiabetic potential. The findings are displayed in Table 5.

The inhibition rates determined for jelly candies with APE were 14.89 ± 0.33% for α-amylase and 15.07 ± 0.67% for β-glucosidase. Therefore, the use of APE in the recipe enriched the jellies with antidiabetic potential. Raczkowska, Wojdyło, and Nowicka (2024) also observed an increase in the antidiabetic potential of shortbread cookies by adding apple pomace in the recipe. More precisely, cookies with 22.7% added apple pomace had a 400-times higher inhibitory activity of α-amylase than that of cookies without apple pomace. Other studies on the inhibitory activity of snacks containing apple pomace against the enzymes mentioned above are not found in the available literature [85]. These results can be correlated with the values obtained for the antioxidant capacity of the prepared jelly candies with AP and the antidiabetic effect of natural antioxidants from fruits or by-products, which have been demonstrated in previous studies [86,87,88,89,90]. These findings are in agreement with the results of the HPLC analysis of phenolic compounds. It was previously reported that protocatechuic acid, the main phenol found in the apple pomace used for jelly preparation, has a significant inhibitory activity against α-amylase [91,92]. The data obtained in this study confirm the hypothesis that apple pomace represents an important antidiabetic source, with available, easy-to-use, and low-cost material, as was also demonstrated by Gorjanović et al. (2020) [86].

### 3.7. Microbiological Studies of Jelly Candies

The results of the microbiological tests (Table 6) show that no colonies of *Enterobacteriaceae* grew on the plates regardless of the presence or absence of apple pomace. Regarding the total number of yeasts and fungi, the samples containing apple pomace exhibited a lower log CFU/g, which remained consistent for up to 7 days of storage compared to the samples without pomace. For instance, at 0, 2, 5, and 7 days of storage, the number of log CFU/g for the samples with apple pomace was, on average, 3.18 log CFU/g, while for the control, at 0 day, the total number of yeasts and fungi was 4.5 log CFU/g; at 2 days, 5.3 log CFU/g; and at 5 and 7 days of storage, the number exceeded 5.3 log CFU/g. According to the Romanian and EU legislation, the maximum limit of yeasts and fungi at the end of the fabrication process is 2 log CFU/g, while for Enterobacteriaceae, it is 0 log CFU/g [93]. Although our results are above the limit for the number of yeasts and fungi, the addition of apple pomace demonstrated its potential to reduce their numbers compared to the jelly without pomace. Antioxidants, known for their antimicrobial properties, may have contributed to the lower number of yeasts and fungi in the samples containing apple pomace [94,95]. Kauser et al. (2024) [9] reported that apple pomace can have antifungal, antibacterial, and antiviral activities due to its content of quercetin glycosides. Also, Teixeira-Lemos et al. (2021) [96] attributed the low number of yeasts and other aerobic fungi in gummy jellies made with natural fruits to their contents in antioxidant compounds. Our results demonstrate that the addition of apple pomace can reduce the number of yeasts and fungi.

### 3.8. Storage Stability of Jelly Candies with/Without Apple Pomace Extract

The stability of the prepared jellies (with and without APE) was tested for a storage period of 7 days at a temperature of 4 °C and in the dark. The jellies were analyzed at T0 and after 2, 5, and 7 days, respectively, in terms of antioxidant capacity, total polyphenol content, and total carotene content. Also, the evolution of the color of the prepared products was evaluated during the stability period.

**Figure 3:**
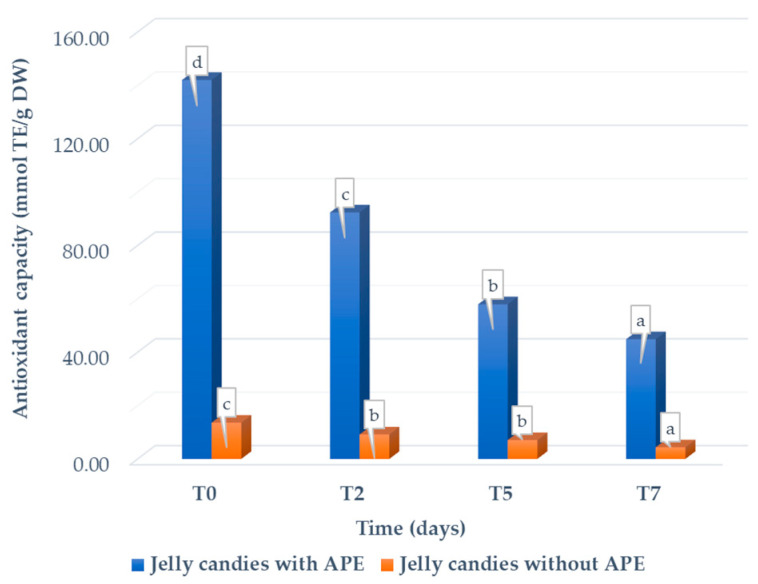
Change in the antioxidant capacity of the jelly candies with and without APE during storage. Different letters of each type of sample (a–d) designate statistically different results (*p* ≤ 0.05).

The antioxidant capacity values recorded during the stability period show a declining trend, both in the jellies with APE and in those without APE (Figure 3). The values decrease significantly for both jelly candies with and without APE, except for the difference between the values obtained for jelly candies without APE in T2 and T5. However, the antioxidant capacity of the jellies with APE remains higher than that of the control jellies. A reduction in the antioxidant capacity of jaboticaba jelly formulations at the end of storage (120 days) was observed by Pinto et al. (2024) [97] and of apple pomace flour jelly, as reported by Gorjanovic et al. (2024) [31].

**Figure 4:**
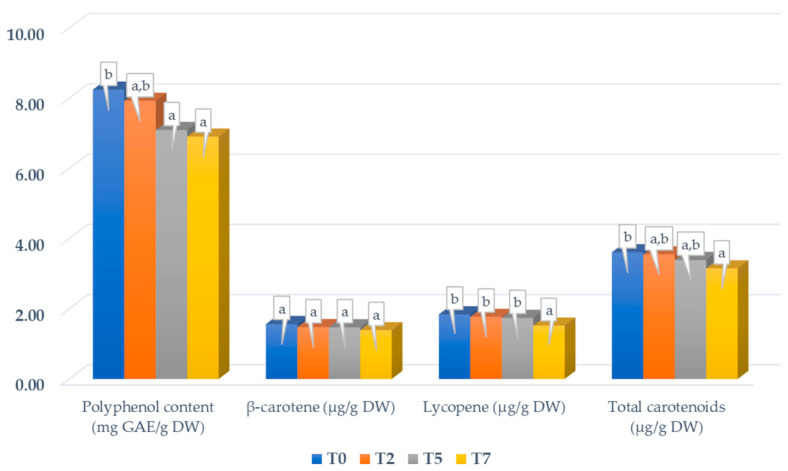
Change in phytochemical content of the jelly candies with APE during storage. Different letters of each parameter (a,b) designate statistically different results (*p* ≤ 0.05).

The same downward trend was observed for the total polyphenol content of jellies with APE (Figure 4). After 7 days of storage, there was a significant decrease of 16.4% compared to T0. This decrease may be the result of the oxidation and polymerization reactions of the polyphenols [90]. Jellies prepared with wine lees [98] and jellies prepared with different blackberry cultivars [99] also showed a degradation in phenolic content over the stability period.

Similar changes were revealed for the jelly candies with APE regarding the carotenoid analysis. More precisely, a decrease of 10.90% was registered for the β-carotene content, 17.39% for the lycopene content, and 12.74% for the total carotenoids after 7 days of storage. The difference between the initial values and those at the end of the storage period was significant for the lycopene and total carotenoid content and was insignificant for the β-carotene content. A previous study on sea buckthorn-grape jellies reported a 50% loss in total carotenoids after 6 months of storage at ambient temperature [100]. The instability of carotenoid compounds is a consequence of their structural features that allow rapid involvement in reactions such as air oxidation and thermal, photo, or acidic degradation [101]. Tobal and Rodrigues [102] also showed a reduction in the antioxidant capacity and bioactive compounds’ content (polyphenols and carotenoids) during storage for both dietary and conventional pitanga fruit jam, highlighting a faster degradation of bioactive compounds in dietary jam.

**Figure 5:**
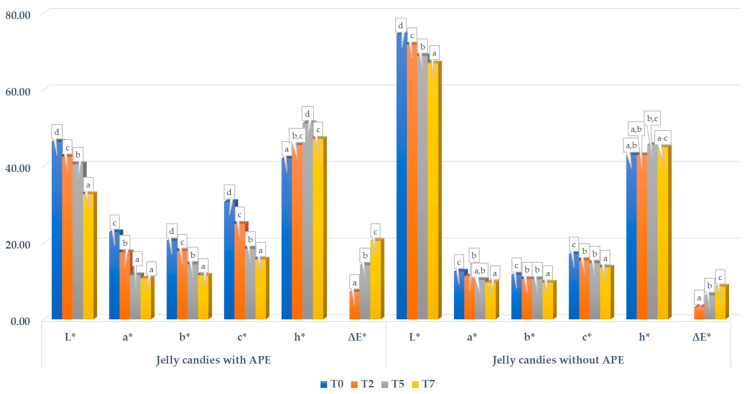
Change in color of the jelly candies with and without APE during storage. Different letters of each parameter (a–d) designate statistically different results (*p* ≤ 0.05).

The measured color parameters L*, a*, and b* slightly decreased during the evaluated period, both for jellies with and without APE (Figure 5). The same tendency was observed for jellies prepared with wine lees [98]. The reduction in the b* values of jellies with APE can be associated with the reduction in carotenoid content during storage.

The overall colorimetric difference, or ΔE*, for each stability evaluation time was calculated against the L*, a*, and b* registered initially (T0). It can be observed that the values increased significantly for both evaluated samples of jellies during storage, with higher differences for jellies with APE. More precisely, after 7 days of storage, the ΔE* of jellies with APE was 20.48 ± 0.37, while for jellies without APE, a value of 8.52 ± 0.16 was calculated.

The data obtained in this stability study are in accordance with results published earlier regarding enriched food products by Enache et al., 2022 [103] (cornelian cherry fruits), Lipșa et al., 2024 [43] (red onion skin), and by Roman et al., 2021 [15] (sea buckthorn fruits).

### 3.9. Sensory Analysis of Jelly Candies

**Figure 6:**
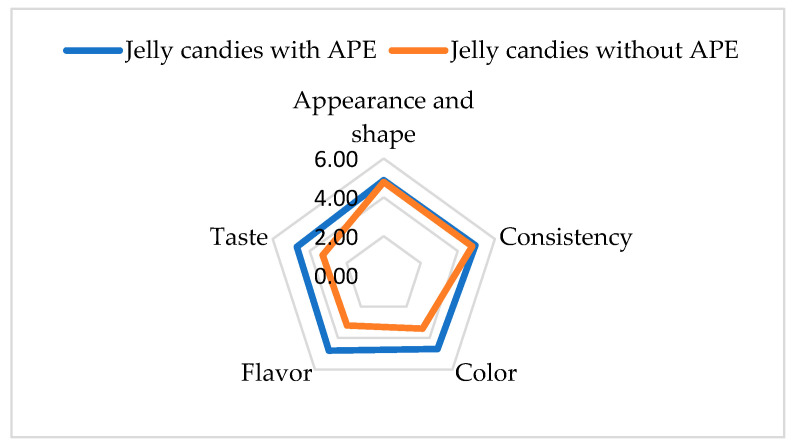
Sensory analysis of jelly candies with/without APE.

A sensory analysis was conducted to assess the consumer acceptance of the proposed jelly candies with APE. The results for both jelly formulations are summarized in Table 7 and are shown graphically in the Figure 6. High scores were obtained for the jelly candies with APE, ranging from 4.70 to 4.95, indicating a sensory-balanced product. Also, it can be observed that jelly candies with APE obtained higher scores than the jelly candies without APE for all the evaluated attributes, with significant differences for color, flavor, and taste. The total organoleptic score of jellies with APE (24.03) was significantly higher than that obtained for jellies without APE (19.47). The evaluators’ positive assessment of color, aroma, and taste characteristics derived from the apple pomace extract proves once again that this ingredient is a healthy natural alternative to consider for synthetic food additives.

## 4. Conclusions

The purpose of this study was to exploit apple pomace, a by-product of the food industry with high amounts of bioactive compounds, in a functional product. The use of APE as the main ingredient in jelly candy formulation led to a product with an improved concentration of bioactive compounds (carotenoids and polyphenols) and an improved antioxidant capacity and antidiabetic potential, as well as balanced sensory characteristics that were highly appreciated by the panelists. In contrast to jellies without APE, jellies with APE were found to be microbiologically stable throughout the evaluation period, showing no colonies of *Enterobacteriaceae* and having the potential to reduce the number of yeasts and fungi. Further studies should be performed to enhance the microbiological profile of the product. All the findings support this study’s hypothesis that jelly candies represent a promising approach for recovering bioactive compounds from apple pomace and emphasize the potential of this by-product to enhance a food product’s quality. The incorporation of APE in jelly candy recipes has been shown to be feasible, bringing a lot of benefits, and no negative effects have been recorded. Also, the technological flow required for producing jelly candies with APE is simple and could be easily replicated in an industrial setting in the future. The superiority of the proposed product over conventional ones is given by its natural flavor and coloring derived from AP, its content of bioactive compounds, and its antioxidant capacity, as well as the absence of sugar or synthetic additives with negative impacts on human health. These encouraging results, obtained for apple pomace as an alternative to synthetic additives in jelly recipes, open new perspectives for the development of other food products. In addition, the successful inclusion of AP as the main ingredient in the recipe of a functional food product contributes to sustainability by reducing waste and using natural resources efficiently. This action aligns with *Goal 12—Responsible consumption and production* of The United Nations’ Sustainable Development Goals.

## Figures and Tables

**Table 1:** Physicochemical profile and color parameters of apple pomace.

Parameter	Apple Pomace Extract
pH	3.20 ± 0.10
Soluble dry matter (°Bx)	4.10 ± 0.01
Total dry matter (%)	3.11 ± 0.03
Total acidity (%)	0.46 ± 0.02
Antioxidant capacity (mmol TE/g DW)	193.26 ± 0.24
Polyphenol content (mg GAE/g DW)	26.94 ± 0.76
β-carotene (µg/g DW)	4.58 ± 0.08
Lycopene (µg/g DW)	4.54 ± 0.04
Total carotenoids (µg/g DW)	10.32 ± 0.12
L*	40.38 ± 0.65
a*	21.21 ± 0.34
b*	20.51 ± 0.28
c*	29.51 ± 0.43
h*	44.04 ± 0.14
Inhibition ratio of α-amylase (%)	32.96 ± 1.72
Inhibition ratio of β-glucosidase (%)	33.32 ± 1.59
Extraction yield (%)	62.50

The results are presented as a mean ± standard deviation.

**Table 2:** The content of the individual phenolic compounds identified in apple pomace.

Crt. No.	Phenolic Compound	Content (µg/g DW)
1.	Protocatechuic acid	375.21 ± 18.76
2.	*p*-Hydroxybenzoic acid	164.96 ± 13.83
3.	Salicylic acid	94.91 ± 0.65
4.	Chlorogenic acid	54.03 ± 4.38
5.	Catechin	37.72 ± 2.84
6.	Vanillic acid	10.55 ± 1.35
7.	Gallic acid	7.94 ± 0.91
8.	Vanillin	6.36 ± 0.82
9.	Coumaric acid	2.65 ± 0.37
10.	Caffeic acid	n.d.
11.	Syringic acid	n.d.
12.	Epicatechin	n.d.
13.	Ferulic acid	n.d.
14.	Sinapic acid	n.d.
15.	Resveratrol	n.d.
16.	Quercetin	n.d.

n.d.—not detected; the results are presented as a mean ± standard deviation.

**Table 3:** Physicochemical profile of jelly candies.

Parameter	Jelly Candies with APE	Jelly Candies Without APE
pH	3.75 ± 0.25 ^a^	4.04 ± 0.01 ^a^
Soluble dry matter (°Bx)	19.00 ± 0.05 ^b^	12.00 ± 0.02 ^a^
Total dry matter (%)	13.64 ± 0.10 ^a^	13.34 ± 0.69 ^a^
Total acidity (%)	0.33 ± 0.01 ^b^	0.08 ± 0.02 ^a^
Antioxidant capacity (mmol TE/g DW)	142.03 ± 1.08 ^b^	13.60 ± 0.58 ^a^
Polyphenol content (mg GAE/g DW)	8.25 ± 0.17	n.a.
β-carotene (µg/g DW)	1.56 ± 0.04	n.a.
Lycopene (µg/g DW)	1.84 ± 0.03	n.a.
Total carotenoids (µg/g DW)	3.61 ±0.28	n.a.

n.a.—not applicable; the results are presented as a mean ± standard deviation, and different letters (^a,b^) designate statistically different results, *p* ≤ 0.05.

**Table 4:** Colorimetric profile of the jelly candies at the initial moment (T0).

Parameter	Jelly Candies with APE	Jelly Candies Without APE
L*	46.42 ± 0.11 ^a^	74.57 ± 0.30 ^b^
a*	22.79 ± 0.49 ^b^	12.47 ± 0.19 ^a^
b*	20.54 ± 0.23 ^b^	11.62 ± 0.36 ^a^
c*	30.68 ± 0.48 ^b^	17.05 ± 0.35 ^a^
h*	42.02 ± 0.45 ^a^	42.96 ± 0.66 ^a^
ΔE*	31.29 ± 0.35 ^#^	-

^#^ overall colorimetric difference between jelly candies with APE and jelly candies without APE; the results are presented as a mean ± standard deviation, and different letters ^(a,b)^ designate statistically different results, *p* ≤ 0.05.

**Table 5:** In vitro evaluation of antidiabetic potential.

Parameter	Jelly Candies with APE	Jelly Candies Without APE
Inhibition ratio of α-amylase (%)	14.89 ± 0.33	n.d.
Inhibition ratio of β-glucosidase (%)	15.07 ± 0.67	n.d.

n.d.—not detected; the results are presented as a mean ± standard deviation.

**Table 6:** The microbiological profile of jelly with and without apple pomace extract.

Sample	Microorganism	Day			
		0	2	5	7
Jelly with APE	Enterobacteriaceae	Absent	Absent	Absent	Absent
	Molds and yeasts	3.3	3	3.2	3.2
Jelly without APE	Enterobacteriaceae	Absent	Absent	Absent	Absent
	Molds and yeasts	4.5	5.3	>5.3	>5.3

Values are expressed in log CFU/g.

**Table 7:** Sensory analysis of jelly candies with/without apple pomace extract (average media).

Sample	Appearance and Shape	Consistency	Color	Flavor	Taste	Total Organoleptic Score
Jelly candies with APE	4.88	4.95	4.70	4.80	4.70	24.03
Jelly candies without APE	4.79	4.78	3.40	3.20	3.30	19.47

## Data Availability

The original contributions presented in this study are included in the article. Further inquiries can be directed at the corresponding author.
